# Fœtid Perspiration of the Feet

**Published:** 1860-07

**Authors:** 


### ARTICLE XVI.

### *Fœtid Perspiration of the Feet.*

MESSRS. EDITORS:—In No. 102 *L'Union Médicale*, a M. Gaffard proposes the following remedy for "fœtid sweating of the feet." Red oxyd of lead, 1 part to 29 parts of the liquor of the subacetate of lead; the first to be bruised in a porcelain mortar, and the liquor gradually added. A few drops to be applied once a week, or oftener, in summer.

This was quoted in the January number of the "*Medical News and Library*" without editorial comment. Possibly, therefore, there may be those who believe in the popular idea that the perspiration excreted on the feet is not inodorous. Professor Hebra, of Vienna, in his lectures on the anatomy and physiology of the skin, has spoken of this notion which is current in Germany. In connection with M. Gaffard's proposed remedy for an evil which certainly does not exist, it may be of interest to quote Prof. H's remarks, which I translate from the notes of his lectures published in the "Allgemeine Wiener Medizinische Zeitung," for 1857.

Respectfully, yours,

B. JOY JEFFRIES, M. D.

15 Chestnut-st., May 2, 1860.

Speaking of the secretion of the perspiration, he says: "There is no doubt that the sweat glands play an important part in the animal economy. Unfortunately, their physiological, and, still more, their pathological relations are but slightly understood. In general, we know that the secretion of sweat is very copious after hard work or continued bodily exertion, especially in the heat; and further, that it is under the influence of the nervous system.

"The sweat is colorless, salt to the taste, has a weak acid reaction, and a peculiar smell. It can scarcely be denied that every individual disseminates a peculiar specific odor. This is proved by dogs following their master's track, and finding him by the help of their greatly developed organ of smell. *Our* organ of smell does not possess the necessary development to enable us to determine such differences. But there are individuals whose peculiar penetrating odor can be easily recognized by every one. It is a great mistake to attribute such a disagreeable smell to the *sweat alone.* We must ascribe it to the secretion of the sebaceous glands. We may be convinced of this by simply examining an individual, the excretions of whose skin have a bad odor. On the palm of the hand, where there are only *sweat* glands, we shall not find any unpleasant smell; it will, on the contrary, be strong on those parts of the body where the sebaceous glands are numerous—as the back, and more particularly in the arm-pits. It is moreover certain that the smell does not come immediately from the *fresh* secretion, but that it exists after this has decomposed. The fresh secretion has either none, or else a slight odor of rancid fat. But if the sweat remains some time in contact with the skin, it undergoes a chemical change, and then the disagreeable smell will be perceived. We will enter more particularly into this subject when we speak of the 'fœtid foot sweat,' which long ago was considered to be a materia peccans whose elimination from the body was desirable, and with whose healthy excretion no therapeutical interference was allowable."

"We come now to speak of a subject upon which similar erroneous views still exist. It is the so-called 'fœtid foot sweat' (bromidrosis.) We have already said that the sweat, when secreted, has no bad odor. Hence it comes that persons troubled with this 'fœtid foot sweat' have no disagreeable odor on the palms of their hands, no matter if the perspiration trickles from them. And when the feet are carefully and properly cleansed (together with the toes and nails,) they lose the highly penetrating smell when they again begin to perspire. This so-called bromidrosis localis is found most frequently in young people, who neglect proper cleanliness, and who possess no superfluity of covering for the feet, so that this is seldom changed. Hence, by the decomposition of the collected sweat, free fat acids are formed that have a disagreeable odor. These are absorbed by the pores of the leather, and one can easily convince himself, through his sense of smell, that the boots are the seat of the odor. Persons wearing a light covering for the feet and often changing it, will have little trouble from 'fœtid foot sweat.' Hence this seldom occurs in the female sex, although the perspiration is more copious in women.

"As, from what has been said, it is evident that we have to deal rather with 'stinking boots' than 'fœtid foot sweat,' the absurd ideas which are in circulation as to the evil effects of suppressing, or too quickly checking the sweating of the feet, must be entirely given up. On other parts of the body also, where the secretion has an opportunity to remain some time in contact with the surface of the skin, *e. g.* in the arm-pits, on the scrotum, perinæum, &c., a similar decomposition of the sweat takes place and a very disagreeable odor is created. The treatment of this 'fœtid sweating of the feet,' is therefore reduced to ordering greater attention to the cleanliness of the skin, and a more frequent changing of the covering of the feet."—*Boston Med. and Surgical Journal.*

